# The impact of semantics on aspect level opinion mining

**DOI:** 10.7717/peerj-cs.558

**Published:** 2021-06-18

**Authors:** Eman M. Aboelela, Walaa Gad, Rasha Ismail

**Affiliations:** Faculty of Computer and Information Sciences, Ain Shams University, Cairo, Egypt

**Keywords:** Opinion mining, Sentiment Analysis, Aspect Lexicon, Wordnet

## Abstract

Recently, many users prefer online shopping to purchase items from the web. Shopping websites allow customers to submit comments and provide their feedback for the purchased products. Opinion mining and sentiment analysis are used to analyze products’ comments to help sellers and purchasers decide to buy products or not. However, the nature of online comments affects the performance of the opinion mining process because they may contain negation words or unrelated aspects to the product. To address these problems, a semantic-based aspect level opinion mining (SALOM) model is proposed. The SALOM extracts the product aspects based on the semantic similarity and classifies the comments. The proposed model considers the negation words and other types of product aspects such as aspects’ synonyms, hyponyms, and hypernyms to improve the accuracy of classification. Three different datasets are used to evaluate the proposed SALOM. The experimental results are promising in terms of Precision, Recall, and F-measure. The performance reaches 94.8% precision, 93% recall, and 92.6% f-measure.

## Introduction

People all over the world use social networking sites to share their experiences, opinions and thoughts on specific topics with others. These opinions and thoughts are an important reference to others in evaluating the products and taking decisions on which product to purchase. Therefore, opinions collection and analysis play a vital role in the product design and marketing. However, the large number of opinions is considered as beneficial as challenging to analyze. Manual analysis is a time consuming and may fail or cause incorrect decisions. Therefore, opinion mining is used to solve this problem by monitoring the peoples’ feelings and emotions toward specific products or services (Arunkarthi & Gandhi, 2016). Opinion mining is useful for filtering the users’ opinions and generating a quick summary from a large amount data in an acceptable time. Thus, the opinion mining can be used in different fields of life, such as commerce, politics, finance, purchasing items, entertainment and others (Singh et al., 2019). Opinion mining consists of three main parts: opinion holder, opinion object, and opinion orientation (Neshan & Akbari, 2020). Opinion holder is the person who gives his/her opinion. Opinion object is the property or aspect that can be expressed by the opinion holder. Opinion polarity is the orientation of the opinionated object as a positive or negative.

Opinion mining uses information retrieval and natural language processing techniques to analyse opinions. Therefore, the opinion mining methods are categorized according to the level of analysis into three levels (Sharma, Nigam & Jain, 2014): document, sentence, and aspect. The document level opinion mining, which classifies the whole document or review with positive or negative based on the number of negative and positive sentiment words that appeared in the review. In this level, the review is assumed to have opinions related to a single object, although the same review may contain different objects with different opinions of the same person. Therefore, this level of analysis is not accurate for decision making. The second level is the sentence-level opinion mining where each sentence is considered as a single review which contains only one opinion. The third one is the aspect level opinion mining. The product aspects are defined as the attributes or properties of the product. The aspects are categorized into implicit and explicit aspects. Implicit aspects are not explicitly written in the review, but extracted from the meaning. Explicit aspects are explicitly located in the review. In aspect level opinion mining, the review is classified into positive and negative label according to the product aspects appeared in the review sentences, whereas the same review may have different aspects with different sentiments. The aspect level opinion mining is the most powerful for decision making as it determines the points of strength and weakness of a product or service.

The aspect level opinion mining has two main steps. The first one is the aspect extraction step where the aspects of the product are extracted from the users’ reviews or comments. The second step is the reviews classification based on the extracted product aspects. Consider the following review from the mobile phone dataset, “the fm radio is cool.” Firstly, “fm radio” is extracted as aspect of mobile phone and (“cool”) as opinion word. Then, the review is classified according to “fm radio” as positive because “cool” is a positive oriented sentiment. The performance of the aspect-based opinion mining process is affected by the presence of negation words and spam aspects which are not related to the product. Therefore, in this paper a semantic-based aspect level opinion mining (SALOM) model is proposed to handle these issues and improve the efficiency of the aspect-based reviews classification. From this perspective, the proposed SALOM does the following: it identifies the actual aspects of the product based on the semantic similarity (Aboelela, Gad & Ismail, 2019). Furthermore, it handles the negation words appeared in the reviews’ sentences to assign accurate orientation scores. The proposed SALOM uses different types of product aspect (nearest aspect synonym, hyponym, and hypernym) to consider more reviews. These aspect types are extracted based on Wordnet taxonomy (Sharma, Nigam & Jain, 2014), and semantic similarity (Wan & Angryk, 2007). SALOM can deal with more than one aspect in the same review sentence. In addition, it uses a sentiment lexicon and two lists of the most popular positive and negative sentiment words to give more accurate sentiment scores. Finally, the proposed model generates a meaningful aspect-based review summary that helps producers and consumers make a decision. The experimental results show a promising performance compared to other methods. SALOM reaches to 94.8%, 93%, and 92.6% in terms of Precision, Recall and F-measure respectively.

The rest of the document is organized like this. Related work in “Related Work” presents a summary of previous studies in the area of opinion mining. How the SALOM model woks are described and explained in “The Proposed Semantic-based Aspect Level Opinion Mining (SALOM) Model”. “Experiments and Results” presents the characteristics of the dataset used, evaluation measures, experiments and outcomes. A discussion can be found in “Discussion”. The conclusion and future work are discussed in “Conclusion and Future Work”.

## Related work

The related work section provides an overview of some studies in the field of opinion mining and sentiment analysis. Two types of studies are given. The first type is linked only to aspect extraction step. The second is related to the opinion mining process at the aspect level (aspect extraction + aspect-based reviews classification).

A pattern knowledge approach is proposed in Htay & Lynn (2013). They defined some patterns to extract the opinionated aspects from the product’s reviews dataset. Nouns and noun phrases are assumed to be product aspects. Examples of the predefined patterns: (adjective, singular noun/plural noun) and (adverb/comparative adverb/superlative adverb, adjective/adverb/comparative adverb/superlative adverb, singular noun/plural noun). Stanford part of speech (POS) tagger (Chinsha & Joseph, 2015) is used to extract the defined patterns from reviews.

Khan, Baharudin & Khan (2014), the authors identified the product aspects by using the semantic relation and the syntactic sequence. The semantic relation is based on polarity adjectives and the syntactic sequence is based on linguistic patterns.

Nouns and noun phrases are considered as product aspects in Maharani, Widyantoro & Khodra (2015). A syntactic pattern approach based on aspects observation is proposed to extract the product aspects from reviews dataset. To extract the aspects that match the predefined patterns, Stanford part of speech (POS) tagger is used. The proposed approach considered the part of speech tag of the current word, previous and next words (current, (current − 1), and (current + 1)).

In Bagheri & Nadi (2018), a framework is proposed to extract the single and multi-word aspects from customer reviews. The authors extracted five products’ datasets from Amazon.com to test the presented framework. The words with noun tags are considered as single-word aspects. For multi-word aspects, several patterns are defined to extract the aspects from reviews’ sentences. Moreover, the c-value measure is calculated for each extracted multi-word aspect to rank the aspects and select the important ones. For each candidate aspect, the Pointwise Mutual Information (PMI) association measure is calculated to extract the final product aspects. Table 1 presents a summary of the studies performed aspect extraction only.

**Table 1:** Summary of studies performed aspect extraction only.

Studies	Advantages	Disadvantages
Htay & Lynn (2013), Khan, Baharudin & Khan (2014), Maharani, Widyantoro & Khodra (2015) and Bagheri & Nadi (2018)	Defined patterns to extract the opinionated product aspects.	The extracted aspects using the defined patterns contained spams. The review classification step is not performed.
Aboelela, Gad & Ismail (2019)	Extracted the accurate product aspects by using semantic similarity measure.	The review classification step is not performed.

In Aboelela, Gad & Ismail (2019), the exact product aspects are extracted by using the WU-Palmer semantic similarity (Wan & Angryk, 2007). Nouns and noun phrases are considered as product aspects. Stanford part of speech (POS) tagger is used for tagging the reviews. The experiments are performed on three different product datasets and achieved promising results in terms of average recall, precision, and f-measure performance measures.

In Hu & Liu (2004), association mining rules and pruning algorithms are applied to find the product aspects from customer reviews. Whereas, the product aspects are assumed to be nouns and noun phrases, and the sentiment words assumed to be adjectives only. The experiments are performed on five products’ datasets collected from the Amazon.com. Wordnet lexicon is used to get the adjectives’ synonyms and antonyms. The negation and but clause are handled.

In Samha, Li & Zhang (2014), an opinionated summary is generated from user reviews. Main four tasks are presented: aspects and opinions extraction and classification of each aspect, grouping aspects based on similarities, find the most popular aspect sentences and finally, generate an opinionated summary. The authors used an opinion lexicon which contained a few numbers of sentiment words and a manually labeled sentiment lexicon to get the sentiment scores of aspects.

In Chinsha & Joseph (2015), adjectives and verbs are used as sentiment words. More combinations are proposed as opinion phrases such as adverbs, adjective combinations, and adverb, verb combinations. Stanford dependency parser (Marneffe, MacCartney & Manning, 2006) is used to extract the aspect related sentiment words from reviews. SentiWordnet (Esuli & Sebastiani, 2006) is used to detect the aspect orientation and Wordnet to get the aspect synonyms. Negative words are handled in classification phase.

Nouns and noun phrases are considered as product aspects in Sarawgi & Pathak (2017). The reviews are extracted from Amazon web services. Natural Language (NL) processor is used for sentence segmentation. Part Of Speech (POS) tagging and Apriori algorithm are used to get the most frequent aspects. Moreover, SentiWordnet lexicon is used to get the sentiment words (adjectives) orientation.

Four modules are presented in Asghar et al. (2017) for aspect-based opinion mining, which namely aspect-sentiment extraction, aspect grouping, aspect-sentiment classification, and aspect-based summary generation. The accuracy of the classification is enhanced by using a sentiment lexicon and a revised corpus. The presented aspect extraction module is an improvement to the work proposed in Khan, Baharudin & Khan (2014). Nouns, noun phrases, and verbs are considered as product aspects. Therefore, an extended set of heuristic patterns is defined to gather the product aspects from dataset reviews. The presented approach achieved the highest precision compared to the other methods in Chinsha & Joseph (2015) and Samha, Li & Zhang (2014). Their approach did not consider the following: extracting the implicit aspects, handling multiple aspects and their related sentiments, slang, and emotions.

A supervised learning model is presented in Haque, Saber & Shah (2018) to classify different unlabeled products’ datasets that come from Amazon.com. The datasets are labeled manually and different classifiers are used to get the right class label. The labeled datasets are processed to extract the aspects of each product. Bag of words, TF-IDF, and chi-square approaches are used to extract the aspects. Different classifiers have also been tested to classify the reviews according to the extracted aspects and to achieve the best classification performance. The used classifiers are the Linear Support Vector Machine, Multinomial Naïve Bayes, Stochastic Gradient Descent, Random Forest, Logistic Regression, and Decision Tree.

In Güner, Coyne & Smit (2019), three different machine learning algorithms are experimented to classify product reviews that come from Amazon.com. The algorithms are the Multinomial Naïve Bayes (MNB), the Linear Support Vector Machine (LSVM), and the Long short-term memory network (LSTM). The experiments showed that the long short-term memory network (LSTM) achieved the highest accuracy. The main tasks of the approach presented in Güner, Coyne & Smit (2019) are aspect extraction, review classification. TF-IDF (Qaiser & Ali, 2018) and tokenization techniques are used for aspects extraction. For the classification with MNB and LSVM, the TF-IDF technique is applied. For classification with LSTM, the tokenization technique is performed. The tokenization technique consisted of three phases which are fitting on the training set, converting text to sequences, and padding sequences. Fitting on the training set was dependent on the number of unique words that existed in the reviews. Converting text to sequences was replacing the review words to unique integers, padding the sequences was making the sequenced arrays are equally sized by adding zeros to arrays.

Two aspect-based methods are proposed in Mowlaei, Abadeh & Keshavarz (2020) to generate dynamic lexicons. The first method used statistical methods and the other one used a genetic algorithm. The dynamic lexicons are context sensitive so, they assigned accurate scores to the words related to the context. The results showed that the use of these dynamic lexicons with most commonly used lexicons such as Bing Liu’s sentiment lexicon, MPQA Subjectivity lexicon, and SentiWordnet achieved better performance than using these known lexicons with each other.

In Neshan & Akbari (2020), a hybrid method is presented which used the lexicon-based techniques along with the machine learning methods to improve the accuracy of reviews classification. Parts of speech tagging is applied and verbs, adjectives and adverbs are considered as sentiment words. Lemmatization is performed for the extracted verbs, adjectives and adverbs and negation words are considered. SentiWordnet Score, positive and negative words ratios, Liu’s lexicon score, and SentiStrength score are used together to calculate a polarity score for each extracted sentiment word. The output polarity scores are used as input of a meta-classifier which classified the reviews to positive and negative based on four machine learning algorithms called Naïve Bayes, Support Vector Machine, Artificial Neural Network, and Decision Table. The results showed that the use of a meta-classifier improved the performance of reviews classification.

Machine learning methods and lexicon-based techniques are used in Keyvanpour, Zandian & Heidarypanah (2020) to enhance the performance of opinions classification. Lexicon-based techniques are applied first and then the machine learning methods. Some documents are considered in the lexicon-based classification such as a document of stop words, a document of emotions along with their orientation, and a document of some positive and negative sentiment words. An enhanced neural fuzzy network is used to improve the performance of the proposed Opinion Mining Method based on Lexicon and Machine Learning (OMLML) method.

Regarding to the mentioned disadvantages in Tables 1 and 2, the previous lexicon-based studies tried to improve the accuracy of reviews classification. However, they did not use the semantic tree to extract the aspects related to the product domain. In addition, they did not consider different types for product aspects. Moreover, most of these studies used only a sentiment lexicon, although it is insufficient to give accurate sentiment scores. Therefore, a semantic-based aspect level opinion mining (SALOM) model is proposed to address these challenges. The proposed model aims to enhance the performance of the opinion mining process and generate a helpful aspect-based review summary.

**Table 2:** Summary of studies performed opinion mining at aspect level.

Studies	Advantages	Disadvantages
Hu & Liu (2004)	Negation is considered.	Only adjectives are assumed as sentiment words. Only frequent aspects are considered. The extracted frequent aspects contained spams.
Samha, Li & Zhang (2014)	An opinion lexicon and a manually built sentiment lexicon are used to assign sentiment scores. The frequent words and their synonyms are considered as product aspects.	Negation is not considered. The extracted aspects set contained spams. The used opinion lexicon consisted of a limited number of opinion words that are assigned scores manually.
Chinsha & Joseph (2015)	Negation is considered. The frequent words and their synonyms are considered as product aspects.	The extracted aspects set contained spams. SentiWordnet lexicon is only used to assign polarity scores.
Sarawgi & Pathak (2017)	Opinion mining at aspect level is performed.	Negation is not considered. Only frequent aspects are considered. Only adjectives are assumed as sentiment words. The extracted frequent aspects contained spams. The only SentiWordnet lexicon is used to assign polarity scores.
Asghar et al. (2017)	Negation is considered. A sentiment lexicon and a revised corpus are used to assign accurate sentiment scores. The semantic similarity measure is applied to group the aspect synonyms.	The extracted aspects by using the defined patterns contained spams.
Haque, Saber & Shah (2018) and Güner, Coyne & Smit (2019)	A supervised learning approach is used at aspect level.	The extracted aspects contained spams.
Mowlaei, Abadeh & Keshavarz (2020)	Negation is considered. The generated dynamic lexicons are used with a group of popular sentiment lexicons to assign accurate sentiment scores.	Only frequent aspects are considered. The generated aspects contained spams.
Neshan & Akbari (2020)	Negation is considered. Lexicon-based techniques are used with machine learning methods to get the reviews polarity.	Aspect extraction step is not considered.
Keyvanpour, Zandian & Heidarypanah (2020)	Lexicon-based techniques are used with machine learning methods to get the aspect-based reviews polarity.	Negation is not considered.

## The proposed semantic-based aspect level opinion mining (salom) model

In this section, we describe our proposed solution, a semantic-based aspect level opinion mining (SALOM) model. The proposed model extracts the actual aspects of the product from reviews based on semantic similarity measure. Then, it classifies the reviews based on different product aspect types. The proposed model consists of two main modules:

- Aspects and related words extraction module.
- Orientation detection module.

**Figure 1:**
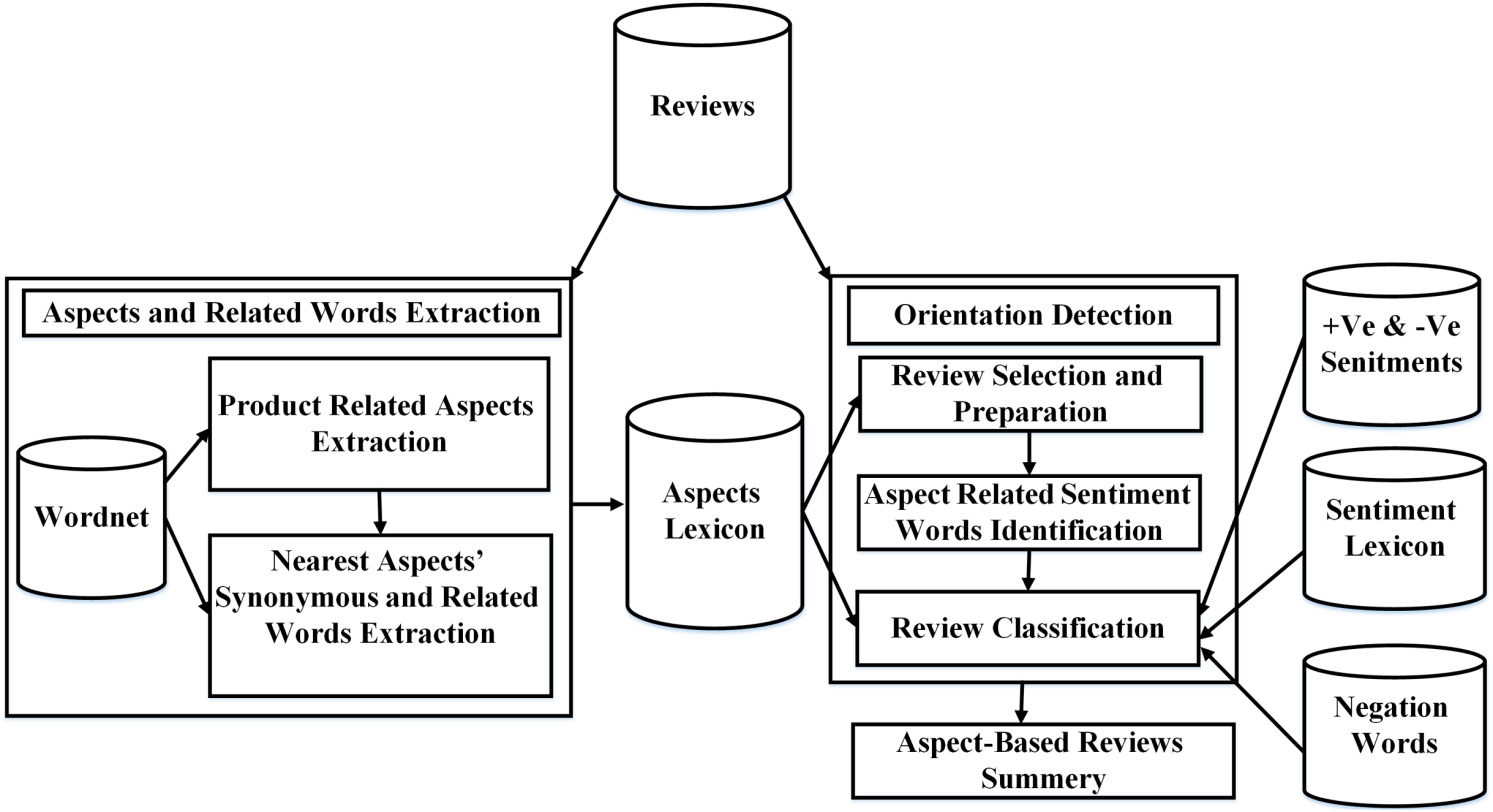
The proposed semantic-based aspect level opinion mining SALOM model.

The proposed SALOM model works as follows: firstly, the most frequented nouns and noun phrases in the dataset are extracted. Then, the semantic similarity is performed to obtain the aspects related to the product domain. The aspect synonyms and related words are important in SALOM. Therefore, for each exact aspect, the nearest synonym, hyponym and hypernym are extracted and considered as product aspects. Whereas, the reviews that have product aspects (actual aspects/aspects’ synonyms/hyponyms/hypernyms) are considered in the classification step. The reviews are preprocessed and tagged to extract the opinion words that express the product aspects. Then, they are classified according to the product aspects into positive or negative based on the polarity of opinion words. Finally, an aspect-based review summary is generated. The overall steps of the proposed SALOM model are shown in Fig. 1 and illustrated in Algorithm 1.

**Algorithm 1:** Semantic-based aspect level opinion mining (SALOM) steps.

**Input:** reviews dataset→*Rds*, Sentiment lexicon→*S_lex*, two lists of positive and negative sentiments
→*Lst_1*, →*Lst_2*, Wordnet lexicon→*Wnet_Lex*, negation set→*Neg_set*, product name→*Prod_nm*.
**Output:** aspect-based reviews summary→*Asp_Sumry*
1: **for each** review *R* in *Rds* **do**
2: Frequented noun and noun phrase aspects *Asp_lst* = Get frequented aspects (Review POS Tags *RPOS*)
3: Actual product aspects *Prd_Asps* = Apply semantic similarity (*Prod]_nm*, *Asp_lst*, *Wnet_Lex*)
4: Nearest Aspect’s synonym, hyponym, and hypernym *Asp_Lex* = Apply semantic similarity
(*Prd_Asps*, *Wnet_Lex*)
5: **end for**
6: **for each** aspect *Asp* in *Asp_Lex* **do**
7: **for each** review *R* in *Rds* **do**
8: Preprocessed Opinionated Review sentence *Op_SentPr* = Get sentence (*R*, *Asp*, *Asp_Lex*)
9: Extracted sentiment words *S_wrds* = Identify sentiment words (*Op_SentPr*, *Asp*, *Asp_Lex*, sentence POS Tags *Op_SentPOS*)
10: *Asp_Sumry[Asp]* = Review classification(*R*, *S_lex*, *Lst_1*, *Lst_2*, *Neg_set*)
11: **end for**
12: **end for**

### Aspects and related words extraction

In this module, product aspect lexicon is built using Wordnet glossary (Sharma, Nigam & Jain, 2014) and semantic similarity. The aspect lexicon consists of the exact product aspects. Each aspect is associated with its nearest synonym, hyponym, and hypernym. In addition, the aspect lexicon is used to filter the product reviews before classification step. Aspects and Related words extraction module consists of:

- Product related aspects extraction.
- Nearest aspects’ synonyms and related words extraction.

#### Product related aspects extraction

The product related aspects extraction step aims to extract the actual product aspects from customers’ reviews. Nouns and noun phrases are assumed to be product aspects. Noun phrases are defined as a sequence of nouns or a combination of adjectives and nouns. Therefore, for each product review, the part of speech (POS) tag is used to extract nouns and noun phrases. For example, the review “the moveable lcd screen is great.” is tagged into (S the/DT moveable/JJ (NP lcd/NN screen/NN) is/VBZ great/JJ. /.) where, the “lcd screen” noun phrase is identified as a candidate product aspect. Then, the frequency is calculated for each extracted noun and noun phrase. The frequency is defined as the number of occurrences of a word. Then, a cutoff threshold is defined to extract the most frequent nouns and noun phrases. Afterwards, the aspects related to the product domain are identified by using the WU-Palmer semantic similarity measure (Wan & Angryk, 2007). The semantic similarity is calculated between the product name and each extracted frequent noun and noun phrase. Then, according to a predefined threshold, the frequent nouns and noun phrases that have the highest semantic similarity score are considered as the actual aspects of the product (Aboelela, Gad & Ismail, 2019). The semantic similarity is calculated using Eq. (1).

(1) $$WUP\_Sim = \displaystyle{{2 \times depth(lcs(c1,c2))} \over {depth(c1) + depth(c2)}}$$

Where, depth (c1, c2) is the depth of the product name sense and each extracted frequent noun and noun phrase sense in the taxonomy. Lcs (c1, c2) is the least common subsume of the senses c1 and c2.

#### Nearest aspects’ synonyms and related words extraction

The nearest aspects’ synonyms and related words extraction step is applied to each exact aspect. Therefore, for each exact product aspect firstly, its synonyms, hyponyms, and hypernyms are extracted using Wordnet glossary. The word synonym is defined in Wordnet as a synset or a set of words (may be a noun or phrase) that share the same meaning of the word. However, hypernym and hyponym of a word; are defined as upper and lower words (nouns or phrases) that are semantically related to the word in the Wordnet hierarchy. Then, the nearest synonym, hyponym, and hypernym of a product aspect are calculated using the WU-Palmer semantic similarity as follows. Firstly, for noun aspects, the nearest synonym to the aspect is measured by calculating the semantic similarity between the aspect, and each word “noun or phrase” exists in its synonym’s bag of words. Then, the word with the greatest semantic similarity score is considered as the nearest synonym to the aspect as shown in Algorithm 2. For example, the synonyms of the word “radio” which is a component or aspect of mobile phone are (‘radiocommunication’, ‘wireless’, ‘radio receiver’, ‘receiving set’, ‘radio set’, ‘tuner’), its hypernyms are (‘broadcasting’, ‘receiver’, ‘receiving system’, ‘communication system’), and its hyponyms are (‘clock radio’, ‘crystal set’, ‘heterodyne receiver’, ‘superheterodyne receiver’, ‘superhet’, ‘push-button radio’, ‘radiotelegraph’, ‘radiotelegraphy’, ‘wireless telegraph’, ‘wireless telegraphy’). After applying the WU-Palmer semantic similarity the nearest synonym, hypernym, and hyponym to the aspect “radio” in the mobile phone domain are ‘radio receiver’, ‘communication system’, and ‘wireless telegraphy’ respectively.

**Algorithm 2:** Nearest aspect synonym, hyponym, and hypernym extraction.

1: **function** GET_WORD_WITH_MAX_SCORE (synonym/hyponym/hypernym bag of words *Lst*,
aspect bag of words *AS_syns*)
**Input:** synonym/hyponym/hypernym bag of words→*Lst*, aspect bag of words→*AS_syns*
**Output:** nearest aspect synonym/hyponym/hypernym→*Nearst_Wrd*
2: *Mx* = −1, *Sim_Score* = 0 : integer variables
3: **for each** Word *W* in *Lst* **do**
4: get synset of *W*→*Word_syns*
5: *Sim_Score* = *AS_syns*.*WUP_Similarity* (*Word_syns*)
6: **if** (*Sim_Score* > *Mx*) **then**
7: *Mx* = *Sim_Score*
8: *Nearst_Wrd* = *W*
9: **end if**
10: **end for**
11: **return** *Nearst_Wrd*
12: **end function**

Secondly, for noun phrase aspects, the aspect is initially divided into words. Then, the nearest synonym for each word in the phrase aspect is identified by applying Algorithm 2. Afterwards, the resulted nearest synonyms of the phrase aspect words are concatenated to get the final nearest synonym to phrase aspect. The same method is applied for hypernym and the hyponym bag of words to find the nearest hyponym and hypernym to the product aspect “noun or noun phrase”.

### Orientation detection

The orientation detection module aims to classify the product reviews to positive label or negative label. The reviews are filtered using the aspect lexicon to distinguish between necessary and unnecessary reviews. Whereas, the reviews that have aspect/aspect synonym/aspect hyponym/aspect hypernym from the aspect lexicon are considered in the classification process. The orientation detection module consists of:

- Review selection and preparation.
- Aspect related sentiment words identification.
- Review classification.

#### Review selection and preparation

The proposed SALOM considers the aspect, aspect synonym, aspect hyponym, and aspect hypernym as product aspects. From this perspective, the review selection and preparation step works as follows. For each review, if the product aspect is not found, the aspect synonym or aspect hyponym or aspect hypernym from the aspect lexicon will be used. So, the review sentence that has the aspect or aspect synonym or aspect hyponym or aspect hypernym is extracted. Then, the unwanted words such as stop words, non-alphabetic characters, and numbers are removed from the sentence.

#### Aspect related sentiment words identification

The proposed semantic-based aspect level opinion mining (SALOM) model assumes the adjectives, adverbs, verbs, nouns, and all their forms are sentiment words. Therefore, to identify the sentiment words that express the product aspects in the review sentences SALOM works as follows.

Initially, the sentence length (the number of words in a sentence) is checked and then it is divided into sized pairs. The pair size is determined according to the aspect length and the sentence length. Therefore, for noun aspects (consist of one word): if the sentence length equals two, then, the pair size is two. However, if the sentence length is greater than two, then, each pair size equals three. But for noun phrase aspects (which consist of two or more words), the sentence length must be greater than two words and each pair size equals (the phrase aspect length + 1). Afterwards, the noun aspect is searched for in all pairs and once it is found, the (POS) tag and the orientation of its associated words in a pair are checked because they are candidates to be sentiment words. However, if the phrase aspect is not found in all pairs, then, it is divided into words and each word is considered as a noun aspect. Algorithm 3 describes how to extract the sentiment words that express the aspect in the sentence.

**Algorithm 3:** Aspect or aspect synonym or aspect hyponym or aspect hypernym related sentiment words extraction.

1: **function** EXTRACT_SENTIMENT_WORDS (preprocessed review sentence *Rev_Sent*, aspect *Asp*)
**Input:** preprocessed review sentence→*Rev_Sent*, aspect →*Asp*
**Output:** list of sentiment words that express the aspect→*SntW_Lst*
2: **if** (length(*Asp*) == 1) and (length (*Rev_Sent*) == 2) **then**
3: Split *Rev_Sent* into pair of (*w*1,*w*2)
4: Extract the aspect from the pair = Search for the aspect in the pair (*Asp*)
5: Check POS and orientation of the word associated with the aspect in pair then append it to *SntW_Lst*
6: **else if** ((length(*Asp*) == 1) or (length(*Asp*) > 1)) and (length(*Rev_Sent*) > 2) **then**
7: **if** (length(*Asp*) == 1) **then**
8: Split *Rev_Sent* into pairs each of (*w*1,*w*2,*w*3)
9: **else**
10: Split *Rev_Sent* into pairs each of size = length(*Asp*)+1
11: Split the aspect *Asp* into words *Ph_wrds*
12: **end if**
13: Extract the pair that contains the aspect *Pr* = Search for the pair (*Asp*)
14: Check POS and orientation of the words associated with the aspect in pair then append them to *SntW_Lst*
15: *if* (length(*Asp*) > 1) and (*Pr* == ””) then
16: **for word** *w* in *Ph_wrds* **do**
17: Extract the pair that contains the word (*w*)
18: Check POS and orientation of the words associated with the aspect in pair then append them to *SntW_Lst*
19: **end for**
20: **end if**
21: **end if**
22: **return** *SntW_Lst*
23: **end function**

#### Review classification

In the proposed (SALOM) model, the review is classified according to each exact product aspect and aspect synonym, hyponym, and hypernym. The polarity of the aspect related sentiment words is detected by using a sentiment lexicon and two auxiliary lists containing the most known positive and negative sentiment words. These auxiliary lists are used in the case that the sentiment lexicon identified the sentiment word as a neutral oriented word.

Two sentiment lexicons “SentiWordnet and Subjectivity lexicon for English adjectives” are used to assign polarity or sentiment scores. The inputs of the two lexicons are different. The SentiWordnet lexicon input is the sentiment word. However, the input of the Subjectivity lexicon for English adjectives is the whole sentence. The experimental results show that the SentiWordnet has better classification performance compared to the subjectivity lexicon. Therefore, SentiWordnet is used in the proposed SALOM model. Moreover, to enhance the accuracy of the review classification and assign more accurate sentiment scores, SALOM considers the negation words that appeared with aspect related sentiment words in the review sentences. Whereas, the existence of these negation words inverts the polarity of the sentiment words. For example, the following two sentences “the vibration is top” and “the vibration is not top” have different orientations. The first sentence is a positive oriented sentence. The negation word “not” in the second sentence reversed the polarity from positive to negative. Therefore, a set of negation word list such as no, not, isn’t, are not, nothing, etc. is used. Algorithm 4 illustrates if the sentiment word is in a negative relation or not. The proposed SALOM model distinguishes between the negative word “not” and “not only”. Whereas, the “not only” word does not invert the orientation.

**Algorithm 4:** Negation handling.

1: **function** CHECK_NEGATION(Parsed sentence *S*, Sentiment word *Op_Wrd*, set of negative words *Neg_Wset*)
**Input:** Parsed sentence→*S*, Sentiment word→*Op_Wrd*, set of negative words→*Neg_Wset*
**Output:** Boolean variable→Flag
2: define *Neg_wrd* = ”” : string variable
3: **for each** Word *W* in *S* **do**
4: **if** (*W* in *Neg_Wset*) **then**
5: *Neg_wrd* = *W*
6: **break**
7: **end if**
8: **end for**
9: **if** ((*Neg_wrd*! = ””) and (*S*.index(*Neg_wrd*) < *S*.index(*Op_Wrd*))) **then**
10: **if** (*Neg_wrd* == “not”) **then**
11: **if** ((“only” in *S*) and ((*S. index*(“only”))−(*S. index*(“not”)) == 1)) **then**
12: Flag = False
13: **else**
14: Flag = True
15: **end if**
16: **else**
17: Flag = True
18: **end if**
19: **end if**
20: **return** Flag
21: **end function**

## Experiments and results

### Dataset description

**Figure 2:**
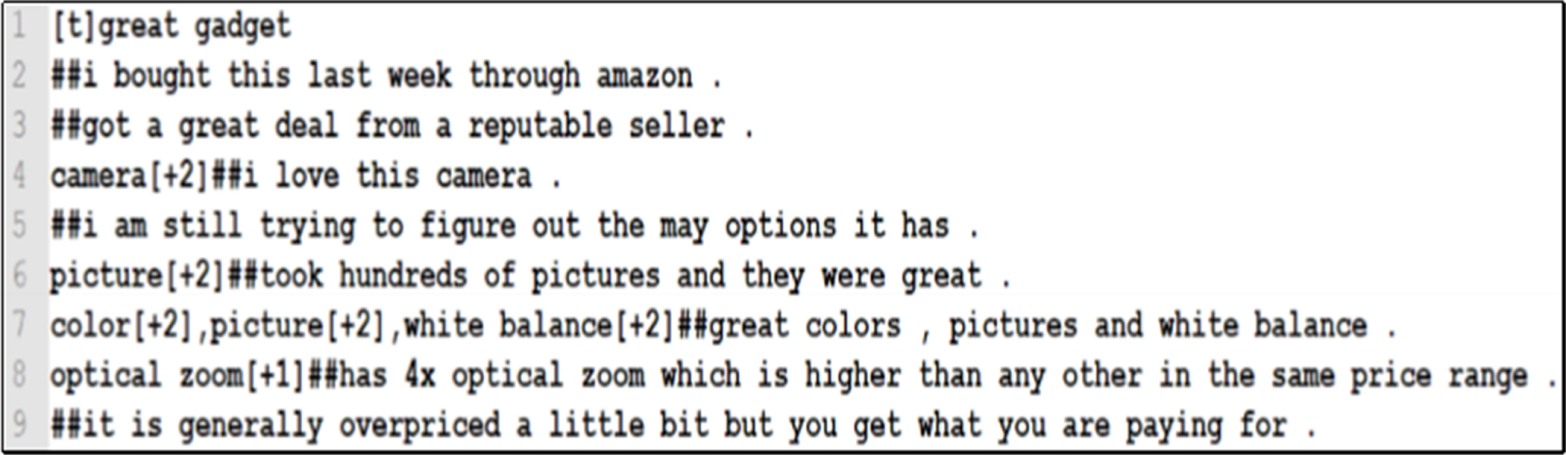
Sample reviews from the digital camera dataset.

To assess the performance of the proposed SALOM model, we experimented three different benchmark Amazon products’ datasets for mobile phone, mp3 player, and digital camera which available on (https://www.cs.uic.edu/ liub/FBS/sentiment-analysis.html). The three datasets are designed, compiled by Hu & Liu (2004) and previously used in Samha, Li & Zhang (2014), Chinsha & Joseph (2015), Asghar et al. (2017), Güner, Coyne & Smit (2019) and Mowlaei, Abadeh & Keshavarz (2020). Table 3 shows the details of each dataset. In the datasets, the reviews are labeled as positive and negative based on their identified aspects. Moreover, they rated from 3 (strongest) to 1 (weakest). Then, the review started after the ## sign. Figure 2 presents sample reviews from the digital camera dataset.

**Table 3:** Details of products’ datasets.

Dataset	#Total reviews	#Reviews associated with a title	#Aspects		Labeled reviews	
			#Implicit	#Explicit	#Pos	#Neg
Digital camera	579	311	32	254	225	61
Mobile phone	546	206	30	310	254	86
Mp3 player	1716	868	116	732	516	332

The datasets have many problems such as:

- Some words are marked as aspects in the dataset but they were not. For example, “8mb card” is labeled as an aspect of the digital camera, but it was not an aspect.
- Some words are defined as aspects in some reviews, but they have not been defined in others, though they are found. For example, “voice dialing” is marked as aspect in some reviews in the mobile phone dataset but not in other reviews.
- The same aspect could have two spellings. For example, the word ringtone had two spellings in the mobile phone dataset “ring tone” and “ringtone”.
- Some aspects are labeled as implicit ones, but they were explicit. For example, the review: “impossibly tiny size and difficult to operate, barely visible, power button”. The “size” aspect has been explicitly defined in the review, but is defined in the dataset as an implicit aspect.
- There are some reviews did not contain labeled aspects, but they were associated with a title as presented in Fig. 2.
- There were some reviews had only implicit aspects.

The proposed (SALOM) model solves the previously mentioned datasets’ problems as follows:

- For those reviews that were associated with a title or not and did not have labeled aspects, the aspects, aspects’ synonyms, hyponyms, and hypernyms are extracted and labeled.
- Reviews that contained implicit aspects and did not explicitly have aspects or aspects’ synonyms/hyponyms/hypernyms from the built aspect lexicon are ignored and considered as spam reviews.
- Each review is analyzed to extract its main aspects that are not mentioned in the dataset and labeled.
- The wrong aspects are deleted.
- The wrong spelling of words is corrected.
- Reviews are divided into sentences.

### Performance measures

Three performance measures called Recall, Precision, and F-measure are used to evaluate the proposed model.

Precision (Pr): is defined as the ratio of correctly classified instances as aspects to the total of positive classified instances.

(2) $$Pr = \displaystyle{{TP} \over {TP + FP}}$$

Recall (R): is defined as the ratio of correctly classified positive instances as aspects to the total classified instances.

(3) $$R = \displaystyle{{TP} \over {TP + FN}}$$

F-measure (Fm): is a metric that combines precision and recall.

(4) $$Fm = 2 \times \displaystyle{{recall \times precision} \over {recall + precision}}$$

In the above formulas, TP (True-Positive) identifies the number of words that are correctly classified as product aspects. FP (False-Positive) identifies the number of words that are in correctly classified as product aspects. FN (False-Negative) identifies the number of words that are incorrectly classified as not product aspects. TN (True-Negative) identifies the number of words that are correctly classified as not product aspects.

### Results and analysis

The proposed semantic-based aspect level opinion mining (SALOM) model is assessed by using the SentiWordnet lexicon and the Subjectivity lexicon for English adjectives. Each lexicon is used with two lists of well-known positive and negative sentiment words. The assessment of the proposed SALOM is based on using different types of aspect: product aspect (frequent and exact), aspect synonym, aspect hyponym, and aspect hypernym as follows:

- F-A: frequent aspects.
- FE-A: frequent exact product aspects by using the WU-Palmer semantic similarity.
- A-ASyns: product aspects and aspects’ synonyms.
- A-ASyns-ARel: product aspects, aspects’ synonyms and Aspects’ related words (hypernyms and hyponyms).

Table 4 shows a comparison between the SentiWordnet lexicon and the Subjectivity lexicon using different types of aspects.

**Table 4:** Comparison between the SentiWordnet lexicon and Subjectivity lexicon using the three products’ datasets.

Lexicon	Aspect types	Datasets								
		Mobile phone			Digital camera			Mp3 player		
		R (%)	Pr (%)	Fm (%)	R (%)	Pr (%)	Fm (%)	R (%)	Pr (%)	Fm (%)
SentiWordNet Lexicon	*F − A*	75	75	74.8	76.7	84.6	78.8	74.8	73.5	72.6
	*FE − A*	98.5	97.2	97.6	81.8	90.4	84	92.7	88.3	88.5
	*A − ASyns*	98.5	97.2	97.6	82.2	90.6	84.5	92.7	89.3	90
	***A − ASyns − ARel***	**98.7**	**97**	**97.7**	**88**	**97.5**	**91.3**	**92**	**90**	**88.8**
Subjectivity Lexicon for English Adjectives	*F − A*	62.8	64.8	62.8	83	62.8	68.3	69.4	67	65.3
	*FE − A*	84.4	71.4	75.5	84.4	71.8	74	63.8	70	64.6
	*A − ASyns*	81.6	74.6	74.7	77	76.3	75.6	72.5	72.7	69
	***A − ASyns − ARel***	**82.6**	**86.8**	**83**	**82.6**	**80**	**80**	**68**	**74.5**	**68.4**

**Note:**

Results of the *A − ASyns − ARel* aspect type for each lexicon are shown in bold.

As shown in Table 4, the performance of the SentiWordnet lexicon and the subjectivity lexicon for English adjectives is improved while considering different kinds of product aspects, whereas for aspect type (*F* − *A*), the two lexicons achieve the lowest classification performance because the aspects set contains spams. However, after the application of semantic similarity and the use of real aspects of the product (*FE* − *A*) the results are improved. Afterwards, the aspect set is extended by another aspect types (*A* − *ASyns*) and (*A* − *ASyns* − *ARel*) to achieve higher performance. As shown in Table 4, some results are not changed after adding a specific aspect type. For example, in mobile phone dataset, results from aspect types (*FE* − *A*) and (*A* − *ASyns*) using the SentiWordnet lexicon are identical. This means that some reviews have neither the aspect nor the aspect synonym.

**Figure 3:**
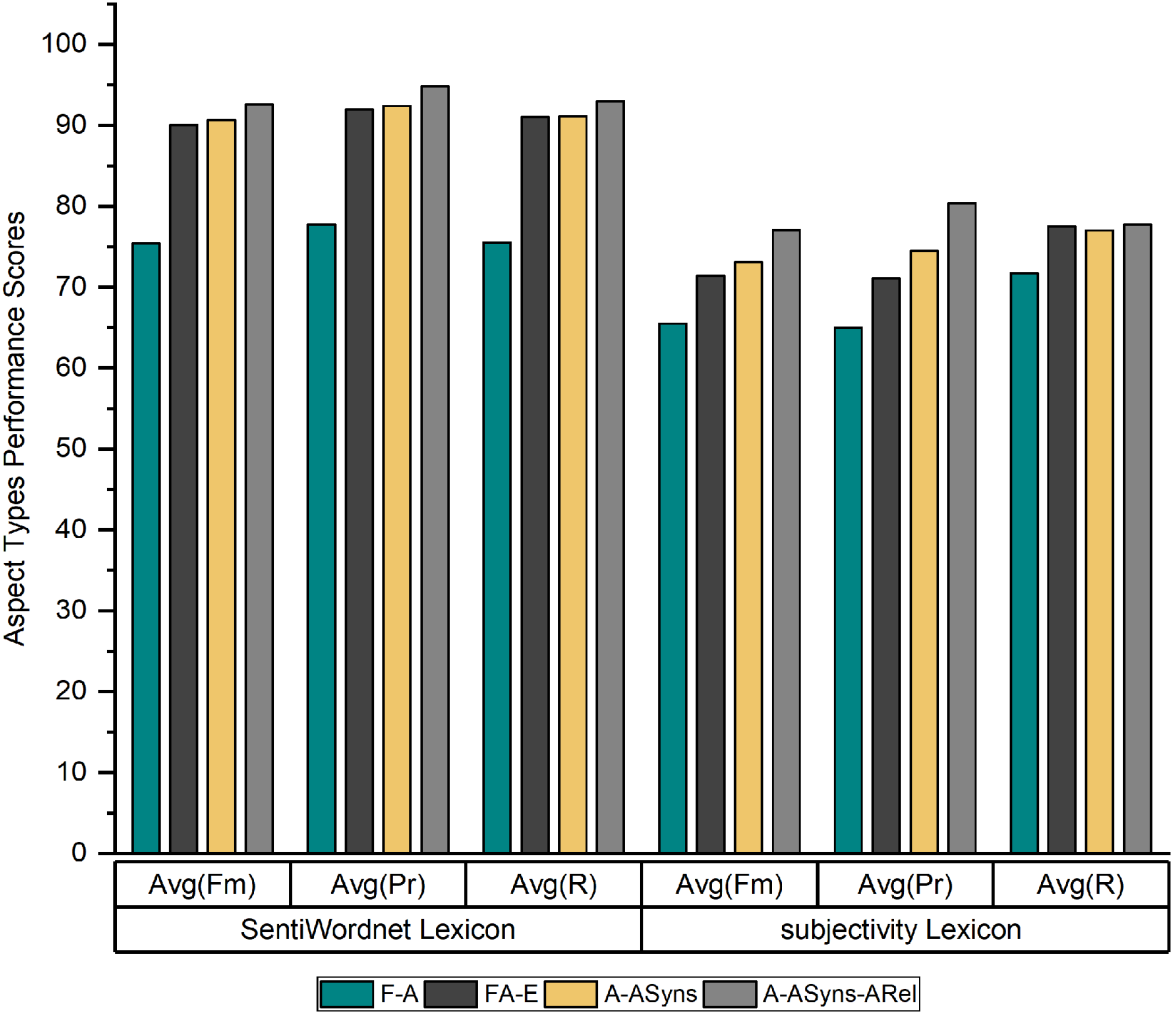
Average recall, precision, and f-measure of the SentiWordnet lexicon and Subjectivity lexicon.

Figure 3 shows the average recall, precision and f-measure of the three products’ datasets by using the SentiWordnet lexicon and the Subjectivity lexicon for English adjectives.

As indicated in Fig. 3, the performance of SALOM using the SentiWordnet lexicon exceeds the performance of the Subjectivity lexicon for English adjectives for (*A* − *ASyns* − *ARel*) aspect type, where the model reaches to 94.8%, 93%, and 92.6% in terms of precision (PR), recall (R), and f-measure (Fm). The reason for this superiority is that the SentiWordnet lexicon considers all part of speech (POS) tags of sentiment words, while the subjectivity lexicon considers only adjective sentiments.

**Figure 4:**
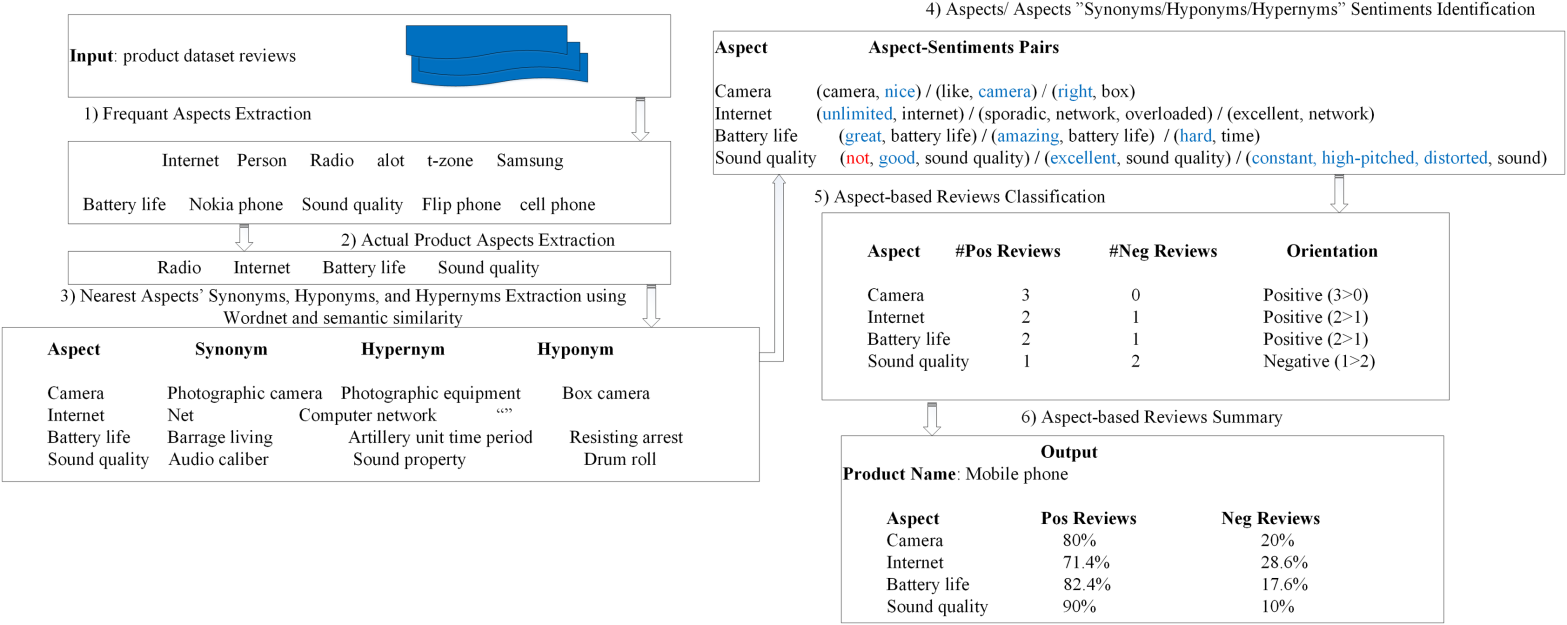
A sample execution example of SALOM model.

A sample execution example of the proposed SALOM model is presented in Fig. 4.

As shown in Fig. 4, the proposed SALOM input is the product reviews dataset. Then, internet, person, radio, alot, t-zone, etc… are extracted from reviews as candidate product aspects. Next, semantic similarity is applied to eliminate the noisier aspects and extract the real aspects of the product. Afterwards, for each aspect of the product, the related synonym, hyponym and hypernym are extracted as indicated in step 3. Then, the aspect related sentiment words are extracted in step 4 where the sentiment word “hard” in pair(hard, time) expresses the “battery life” aspect. This means that, for a particular review, SALOM finds neither the aspect “battery life” nor its separate words “battery” and “life” nor the aspect synonym “Barrage living” nor its separate words nor the aspect hypernym “Artillery unit time period”. However, SALOM finds the aspect hypernym word “time” and extracts its sentiment word “hard”. Moreover, the sentiment word “right” in the pair (right, box) expresses the aspect “camera” because the word “box” comes from the camera hyponym “camera box”. In step 5, for each aspect of the product, the number of positive and negative reviews is determined. According to the “sound quality” aspect in the pair (not, good, sound quality), The existence of the negation word “not” changes its polarity from positive to negative. Finally, the aspect-based review summary is generated as stated in step 6.

A comparison between the proposed SALOM model and other methods in Hu & Liu (2004), Samha, Li & Zhang (2014), Chinsha & Joseph (2015), Asghar et al. (2017), Güner, Coyne & Smit (2019) and Mowlaei, Abadeh & Keshavarz (2020) is presented in Table 5 to show how each of them extracts the product aspects.

**Table 5:** A comparison between the proposed SALOM model and other methods for aspect extraction.

Reference	Method
Hu & Liu (2004)	Association mining rules are used to extract the frequent nouns and noun phrases (recommended to be product aspects). Then, some pruning techniques are applied to eliminate the meaningless frequent aspects and extract the actual product aspects.
Samha, Li & Zhang (2014)	Initially a list of predefined product specifications/aspects and their synonyms is considered. Then, the part of speech tagging is performed to extract the frequent tag sets from customer reviews. Finally, the product aspects are extracted from the frequent tags based on the list of product specifications and their synonyms.
Chinsha & Joseph (2015)	Part of speech tagging is applied to get the frequent noun and noun phrase words. Then, unneeded words are removed and synonyms words are grouped to generate the final product aspects.
	Furthermore, the aspects’ synonyms are considered as product aspects.
Asghar et al. (2017)	An expanded patterns set than the one proposed by Khan, Baharudin & Khan (2014) is used to extract the product aspects. Whereas, the patterns considered nouns, noun phrases, and verbs as product aspects.
Güner, Coyne & Smit (2019)	Two techniques called TF-IDF Vectorizer and tokenization technique are presented for product aspect extraction. The type of classifier used to determine the technique used to obtain aspects of the product. The tokenization technique involved three steps which are (1) getting the maximum number of reviews unique words, (2) converting each review into array of unique integer numbers, and (3) making the arrays equally sized.
Mowlaei, Abadeh & Keshavarz (2020)	Predefined part of speech tags are used to extract words that are candidates to be product aspects.
	Each extracted word assigned a score, which depended on the following considerations: (1) The frequency of the word in positive and negative sentences, (2) The number of positive and negative aspects in the dataset, and (3) The distance between the word and the existing aspects in the training corpus. The words with maximum scores are considered to be product aspects.
Proposed SALOM	Part of speech tagging is applied to extract the most frequent nouns and noun phrases. Then, semantic similarity is performed to extract the actual product aspects. Moreover, the closest synonym, hypernym, and hyponym to aspects are considered as product aspects.

Table 6 shows a performance comparison between the proposed semantic-based aspect level opinion mining (SALOM) model and the methods presented in Hu & Liu (2004), Samha, Li & Zhang (2014), Chinsha & Joseph (2015), Asghar et al. (2017), Güner, Coyne & Smit (2019) and Mowlaei, Abadeh & Keshavarz (2020).

**Table 6:** Performance comparison between the proposed SALOM model and other methods.

Methods	Performance measures		
	Avg.R (%)	Avg.Pr (%)	Avg.Fm (%)
Hu & Liu (2004)	69.3	64.2	84.2
Samha, Li & Zhang (2014)	61	56	60
Chinsha & Joseph (2015)	90.1	79.75	78
Asghar et al. (2017)	73	85	78
Güner, Coyne & Smit (2019)	55.5	64	61.4
Mowlaei, Abadeh & Keshavarz (2020)	91.0	91.0	91.0
**SALOM**	**93**	**94.8**	**92.6**

**Note:**

The SALOM measures are shown in bold.

As shown in Table 6, the proposed model achieves better performance compared to other methods in terms of Recall, Precision and F-measure respectively. As, the proposed SALOM model uses the semantic similarity to avoid spam product aspects. In addition, it uses different types of product aspect, such as aspect synonym, hyponym, and hypernym which also related to the product domain. Moreover, SALOM handles the negation words and differentiates between the negation word “not” and “not only”. Finally, SALOM can deal with different aspects exist in the same review sentence.

## Discussion

The proposed SALOM is assessed using four experiments to discover the effectiveness of semantics in the opinion mining process. The experiments are based on the SentiWordnet lexicon, which achieved better performance than the subjectivity lexicon. Whereas, the SentiWordnet lexicon targets all the words, regardless their part of speech tags, unlike the subjectivity lexicon, that targets adjectives only. To give more accurate sentiment scores, the SentiWordnet is used with two lists of most known negative and positive sentiments. The first experiment used the frequently mentioned words in the dataset as product aspects. However, SALOM achieved low precision, recall, and f-measure because of spam words that are considered as product aspects. Therefore, in the second experiment, the semantic similarity is used to find the exact aspects related to the product domain, and the results are improved. To increase the performance improvement, the third and fourth experiments used the exact aspects, and their nearest synonyms and related words (hyponyms and hypernyms) as product aspects. The nearest aspects synonyms and related words are extracted using Wordnet glossary and semantic similarity. Furthermore, handling the negation words in the four experiments improved the accuracy of reviews classification at aspect level. The results showed that the use of semantic similarity, other types of the product aspects, and handling the negation have a great impact on the performance of aspect-based opinion mining process.

## Conclusion and future work

In this paper, a semantic-based aspect level opinion mining (SALOM) model is proposed. The proposed model used the WU-Palmer semantic similarity measure to extract the exact product aspects. SALOM assumed an aspect as a noun or noun phrase. It classified the reviews based on different aspect types: exact product aspects, aspects’ synonyms, hyponyms, and hypernyms. The proposed SALOM considered the negation words in the classification step. Two sentiment lexicons are used: SentiWordnet lexicon and Subjectivity lexicon for English Adjectives. The experimental results showed that the SentiWordnet outperformed the Subjectivity lexicon. Therefore, the experiments were based on the SentiWordnet lexicon. SALOM outperformed the other methods and achieved 94.8%, 93%, and 92.6% in terms of Precision, Recall, and F-measure performance measures.

Several improvements to this research that will be discussed in the future. The first improvement involves grouping together aspect synonyms such as “battery” and “battery life” will be grouped in “battery”. The second is to deal with the implicit aspects that are extracted according to the context of the review. Finally, considering the strength of opinion words and proposing a new method to assign more specific sentiment scores are other improvements.

## Supplemental Information

10.7717/peerj-cs.558/supp-1 Supplemental Information 1 SALOM code.

## Additional Information and Declarations

### Data Availability

The following information was supplied regarding data availability: A Python file and two Notepad files are available in the Supplemental File. Three different benchmark Amazon products’ datasets for mobile phone, mp3 player, and digital camera are available at https://www.cs.uic.edu/˜liub/FBS/sentiment-analysis.html.
